# Supplementary material for: Non-predictive online spatial coding in the posterior parietal cortex when aiming ahead for catching
Source: Sci Rep. 2018 May 17;8:7756. doi: 10.1038/s41598-018-26069-1 (PMC5958121; doi:10.1038/s41598-018-26069-1)
Supplement: Supplementary file 1 — Supplementary Information [file 41598_2018_26069_MOESM1_ESM.pdf]

SUPPLEMENTARY INFORMATION

Non-predictive online spatial coding in the posterior parietal cortex when aiming ahead for catching

Sinéad A. Reid[1] & Joost C. Dessing*[,1]

1: School of Psychology, Queen's University Belfast, David Keir Building, 18-30 Malone Road, BT9 5BN Belfast, Northern Ireland

* j.dessing@qub.ac.uk, Tel: 0044-28-90975650

Supplementary Fig. 1

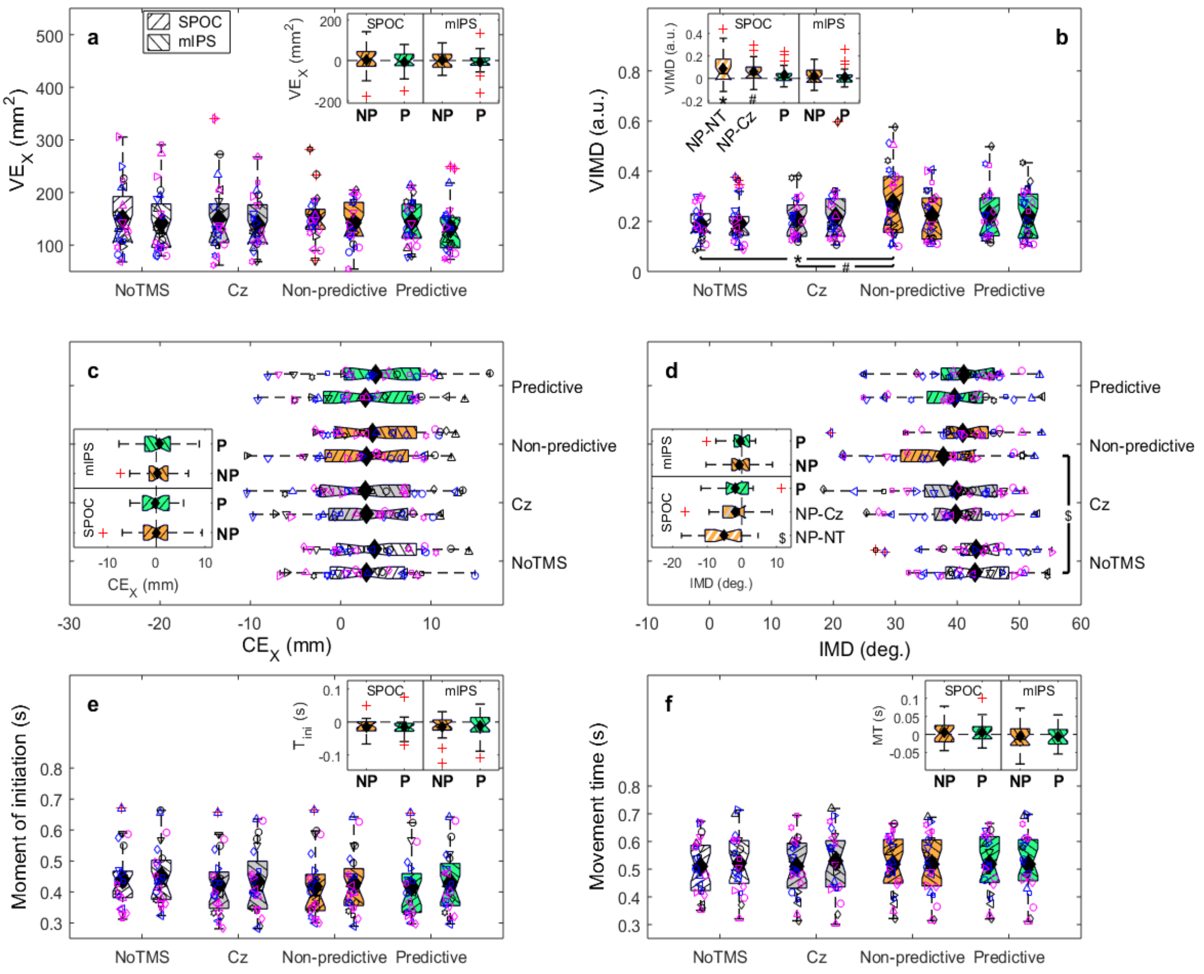

Supplementary Figure 1 Standard boxplots for the Non-predictive and Predictive rTMS effects on all horizontal movement variability and biases and timing for SPOC and mIPS. (**a**) Shows the effects on the variability of the horizontal interception error (VE_X). (**b**) Shows the effects on the variability of the initial movement direction (VIMD). (**c**) Shows the effects on the horizontal interception error (CE_X). (**d**) Shows the effects on the initial movement direction (IMD). (**e**) Shows the effects on the moment of initiation (T_{ini}). (**f**) Shows the effects on the movement time (MT). In all panels, the Non-predictive (NP) and Predictive (P) rTMS effects are shown relative to rTMS to the control site Cz and the condition without rTMS (NoTMS, NT). Insets show the Non-predictive (**NP** = NP-(NT+Cz)/2) and Predictive (**P** = P-(NT+Cz)/2) contrasts; for significant contrasts the individual comparisons with NoTMS and Cz are shown instead. In the main panels, individual data is depicted using unique symbol/colour/jitter combinations. Means are indicated by filled diamonds; outliers are red crosses. *: $t(23) = 3.08$, $p = 0.0026$, $d' = 0.63$, $\alpha = 0.00625$; #: $t(23) = 3.26$, $p = 0.0017$, $d' = 0.67$, $\alpha = 0.00313$ (one-tailed paired-samples *t*-tests). $: $\chi^2(1) = 12.77$; $p = 0.00035$, $\alpha = 0.00313$ (main contrast: $\chi^2(1) = 8.78$; $p = 0.0031$, $\alpha = 0.00625$) (circular likelihood ratio tests [relative to 0][32]). Validity of results (*p*-values) confirmed using 1,000,000 bootstraps.

Supplementary Fig. 2

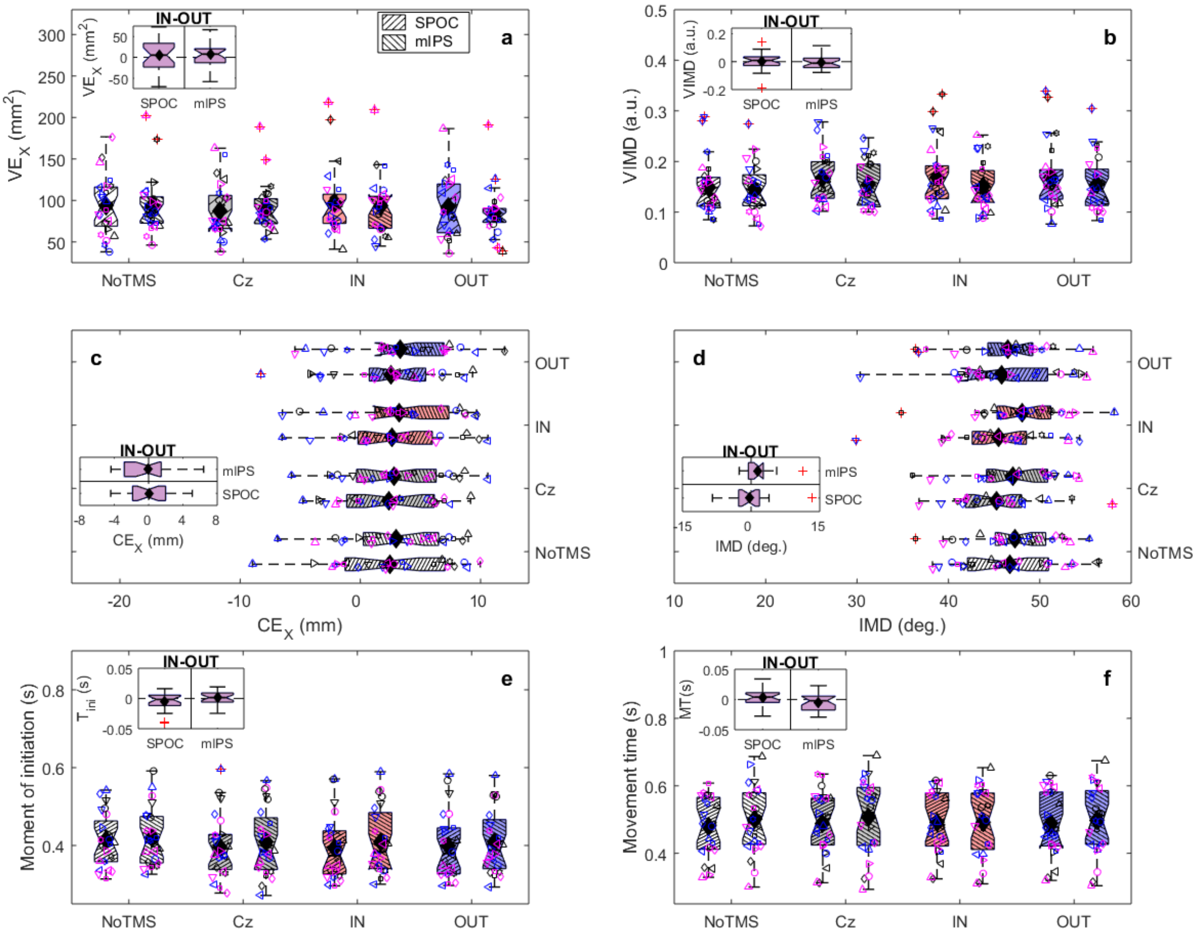

Supplementary Figure 2 Standard boxplots for the rTMS effects for non-diagonal target trajectories for both SPOC and mIPS. (**a**) Effects for the variable error of the horizontal interception error (VE_X). (**b**) Effects for the variability of initial movement direction (VIMD). (**c**) Effects for the horizontal interception error (CE_X). (**d**) Effects for the initial movement direction (IMD). (**e**) Effects for the moment of initiation (T_{ini}). (**f**) Effects for the movement time (MT). Insets show the contrasts between the rTMS effects for targets that, according to their retinotopic position, would be coded inside (IN) the stimulated hemisphere or in the opposite hemisphere (OUT). None of the differences were significant. Validity of results (*p*-values) confirmed using 1,000,000 bootstraps.

Supplementary Fig. 3

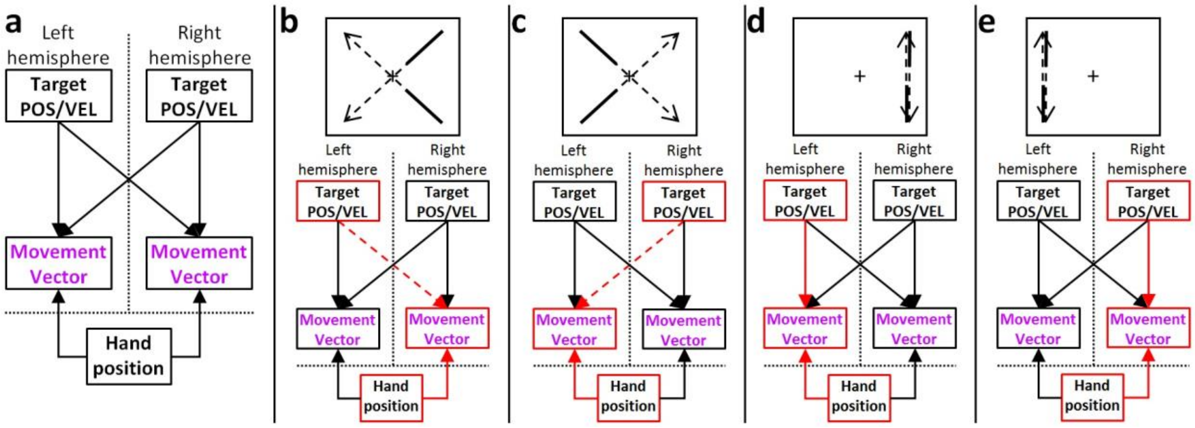

Supplementary Figure 3 A schematic of hemisphere-specific movement planning. (**a**) Calculation of the movement vector in each hemisphere, based on combined target position and velocity signals (target POS/VEL) – originating in SPOC according to the interpretation of our findings – and hand position signals. Any hemisphere specificity of the latter is omitted because it is not pertinent to our interpretation. (**b**) The active nodes and pathways for diagonal target trajectories from the right to the left visual hemifield. (**c**) The active nodes and pathways for diagonal target trajectories from the left to the right visual hemifield. (**d**) The active nodes and pathways for non-diagonal target trajectories in the right visual hemifield. (**e**) The active nodes and pathways for non-diagonal target trajectories in the left visual hemifield. In all cases the target's position and velocity are coded in the hemisphere opposite to the visual hemifield containing the current target position. For diagonal trajectories ((**b**)&(**c**)), the movement vector is computed in the hemisphere opposite to the visual hemifield containing the *final* target position. For straight trajectories ((**d**)&(**e**)) the movement vector is coded in the hemisphere opposite to the visual hemifield containing the *current* target position. Our interpretation depends on rTMS predominantly affecting signals from SPOC that cross to the opposite hemisphere, which are displayed as dashed arrows in (**b**) and (**c**). For simplicity and lack of evidence, a stage explicitly coding the predicted future target position (using target position and velocity signals from SPOC) is omitted; our results would suggest that SPOC does *not* represent this stage.

Supplementary Table 1: Non-predictive and Predictive rTMS effects on spatial movement variability

Site/DV	Test	Statistics	$p_{bootstrapped}$
SPOC/VE_X	Non-predictive	vs. NoTMS: $t(23)=0.47$; $p=0.32$; $\alpha=0.025$; $d'=0.096$<br>vs. Cz: $t(23)=0.48$; $p=0.32$; $\alpha=0.0126$; $d'=0.098$	$p=0.32$<br>$p=0.32$
	Predictive	vs. NoTMS: $t(23)=-0.19$; $p=0.57$; $\alpha=0.025$; $d'=-0.039$<br>vs. Cz: $t(23)=-0.14$; $p=0.56$; $\alpha=0.0126$; $d'=-0.029$	$p=0.58$<br>$p=0.56$
	(Non-predictive - (NoTMS+Cz)/2) UP vs. DWN	$t(23)=-1.28$; $p=0.21$; $\alpha=0.00625$; $d'=-0.26$	$p=0.19$
	(Predictive - (NoTMS+Cz)/2) UP vs. DWN	$t(23)=-0.30$; $p=0.77$; $\alpha=0.00625$; $d'=-0.061$	$p=0.76$
	(Non-predictive - (NoTMS+Cz)/2) L vs. R	$t(23)=-0.47$; $p=0.65$; $\alpha=0.00625$; $d'=-0.095$	$p=0.64$
	(Predictive - (NoTMS+Cz)/2) L vs. R	$t(23)=-0.0019$; p >0.99; $\alpha=0.00625$; d'=-0.00038	$p > 0.99$
mIPS/VEx	Non-predictive	$t(23)=0.32$; $p=0.38$; $\alpha=0.00625$; $d'=0.065$	$p=0.37$
	Predictive	$t(23)=-0.71$; $p=0.76$; $\alpha=0.00625$; $d'=-0.15$	$p=0.77$
	(Non-predictive - (NoTMS+Cz)/2) UP vs. DWN	$t(23)=-0.26$; $p=0.80$; $\alpha=0.00625$; $d'=-0.052$	$p=0.79$
	(Predictive - (NoTMS+Cz)/2) UP vs. DWN	$t(23)=0.49$; $p=0.63$; $\alpha=0.00625$; $d'=0.10$	$p=0.62$
	(Non-predictive - (NoTMS+Cz)/2 L vs. R	$t(23)=1.36$; $p=0.19$; $\alpha=0.00625$; $d'=0.28$	$p=0.16$
	(Predictive - (NoTMS+Cz)/2) L vs. R	$t(23)=0.75$; $p=0.46$; $\alpha=0.00625$; $d'=0.15$	$p=0.45$
SPOC/VIMD	Non-predictive	**$t(23)=3.45$; $p=0.0011$; $\alpha=0.00625$; $d'=0.70$**<br>**vs. NoTMS: $t(23)=3.08$; $p=0.0026$; $\alpha=0.00625$; $d'=0.63$**<br>**vs. Cz: $t(23)=3.26$; $p=0.0017$; $\alpha=0.00313$; $d'=0.65$**	**$p=0.00045$**<br>**$p=0.0018$**<br>**$p=0.00092$**
	Predictive	$t(23)=1.75$; $p=0.046$; $\alpha=0.00625$; $d'=0.36$	$p=0.044$
	(Non-predictive - (NoTMS+Cz)/2) UP vs. DWN	$t(23)=0.47$; $p=0.64$; $\alpha=0.00625$; $d'=0.096$	$p=0.63$
	(Predictive - (NoTMS+Cz)/2) UP vs. DWN	$t(23)=-0.17$; $p=0.87$; $\alpha=0.00625$; $d'=-0.035$	$p=0.86$
	(Non-predictive - (NoTMS+Cz)/2) L vs. R	$t(23)=-0.20$; $p=0.84$; $\alpha=0.00625$; $d'=-0.040$	$p=0.84$
	(Predictive - (NoTMS+Cz)/2) L vs. R	$t(23)=0.26$; $p=0.80$; $\alpha=0.00625$; $d'=0.052$	$p=0.80$
mIPS/VIMD	Non-predictive	vs. NoTMS: $t(23)=1.67$; $p=0.055$; $\alpha=0.0126$; $d'=0.34$<br>vs. Cz: $t(23)=0.27$; $p=0.39$; $\alpha=0.025$; $d'=0.056$	$p=0.047$<br>$p=0.41$
	Predictive	vs. NoTMS: $t(23)=-1.44$; $p=0.082$; $\alpha=0.0126$; $d'=0.29$<br>vs. Cz: $t(23)=0.10$; $p=0.46$; $\alpha=0.025$; $d'=0.021$	$p=0.076$<br>$p=0.46$
	(Non-predictive - (NoTMS+Cz)/2) UP vs. DWN	$t(23)=-1.12$; $p=0.27$; $\alpha=0.00625$; $d'=-0.23$	$p=0.25$
	(Predictive - (NoTMS+Cz)/2) UP vs. DWN	$t(23)=-0.0072$; $p=0.99$; $\alpha=0.00625$; $d' = -0.0015$	$p=0.99$
	(Non-predictive - (NoTMS+Cz)/2) L vs. R	$t(23)=0.24$; $p=0.82$; $\alpha=0.00625$; $d'=0.048$	$p=0.81$
	(Predictive - (NoTMS+Cz)/2) L vs. R	$t(23)=0.43$; $p=0.67$; $\alpha=0.00625$; $d'=0.087$	$p=0.66$

Note: rTMS=repetitive Transcranial Magnetic Stimulation; DV=Dependent variable; SPOC=Superior Parietal Occipital Cortex; mIPS=Medial Intraparietal Sulcus; VE_X=variance of horizontal interception error; VIMD: variability of initial movement direction; NoTMS=conditions without rTMS; Cz=conditions with rTMS applied to the control site Cz; UP=upward target motion; DWN=downward target motion; L=stimulation applied in the left hemisphere; R=stimulation applied in the right hemisphere.

Supplementary Table 2: Non-predictive and Predictive rTMS effects on spatial movement biases

Site/DV	Test	Main contrast	Bootstrapped *p*
SPOC/CE$_X$	Non-predictive	$t(23)=-0.072$; $p=0.94$; $\alpha=0.00625$; $d'=-0.015$	$p=0.94$
	Predictive	$t(23)=-0.19$; $p=0.85$; $\alpha=0.00625$; $d'=-0.04$	$p=0.84$
	(Non-predictive - (NoTMS+Cz)/2) UP vs. DWN	$t(23)=0.71$; $p=0.49$; $\alpha=0.00625$; $d'=0.14$	$p=0.47$
	(Predictive - (NoTMS+Cz)/2) UP vs. DWN	$t(23)=-1.65$; $p=0.11$; $\alpha=0.00625$; $d'=-0.34$	$p=0.091$
	(Non-predictive - (NoTMS+Cz)/2) L vs. R	$t(23)=0.80$; $p=0.43$; $\alpha=0.00625$; $d'=0.16$	$p=0.42$
	(Predictive - (NoTMS+Cz)/2) L vs. R	$t(23)=-1.03$; $p=0.32$; $\alpha=0.00625$; $d'=-0.21$	$p=0.29$
mIPS/CEx	Non-predictive	$t(23)=0.36$; $p=0.72$; $\alpha=0.00625$; $d'=0.074$	$p=0.71$
	Predictive	$t(23)=0.80$; $p=0.43$; $\alpha=0.00625$; $d'=0.16$	$p=0.41$
	(Non-predictive - (NoTMS+Cz)/2) UP vs. DWN	$t(23)=0.11$; $p=0.92$; $\alpha=0.00625$; $d'=0.022$	$p=0.91$
	(Predictive - (NoTMS+Cz)/2) UP vs. DWN	$t(23)=-1.61$; $p=0.12$; $\alpha=0.00625$; $d'=-0.33$	$p=0.099$
	(Non-predictive - (NoTMS+Cz)/2 L vs. R	$t(23)=0.14$; $p=0.89$; $\alpha=0.00625$; $d'=0.029$	$p=0.88$
	(Predictive - (NoTMS+Cz)/2) L vs. R	$t(23)=0.54$; $p=0.60$; $\alpha=0.00625$; $d'=0.11$	$p=0.58$
SPOC/IMD	Non-predictive	**$\chi^2(1)=8.78$; $p=0.0031$; $\alpha=0.00625$**<br>**vs. NoTMS: $\chi^2(1)=12.77$; $p=0.00035$; $\alpha=0.00313$**<br>vs. Cz: $\chi^2(1)=2.61$; $p=0.11$; $\alpha=0.00625$	**$p=0.00067$**<br>**$p=0.000017$**<br>$p=0.086$
	Predictive	$\chi^2(1)=2.79$; $p=0.095$; $\alpha=0.00625$	$p= 0.076$
	(Non-predictive - (NoTMS+Cz)/2) UP vs. DWN	$\chi^2(1)=0.0011$; $p=0.97$; $\alpha=0.00625$	$p=0.96$
	(Predictive - (NoTMS+Cz)/2) UP vs. DWN	$\chi^2(1)=0.0029$; $p=0.96$; $\alpha=0.00625$	$p=0.96$
	(Non-predictive - (NoTMS+Cz)/2) L vs. R	$\chi^2(1)=0.79$; $p=0.32$; $\alpha=0.00625$	$p=0.30$
	(Predictive - (NoTMS+Cz)/2) L vs. R	$\chi^2(1)=0.26$; $p=0.61$; $\alpha=0.00625$	$p=0.61$
mIPS/IMD	Non-predictive	$\chi^2(1)=0.41$; $p=0.52$; $\alpha=0.00625$	$p=0.51$
	Predictive	$\chi^2(1)=0.30$; $p=0.58$; $\alpha=0.00625$	$p=0.57$
	(Non-predictive - (NoTMS+Cz)/2) UP vs. DWN	$\chi^2(1)=0.39$; $p=0.53$; $\alpha=0.00625$	$p=0.52$
	(Predictive - (NoTMS+Cz)/2) UP vs. DWN	$\chi^2(1)=1.08$; $p=0.30$; $\alpha=0.00625$	$p=0.27$
	(Non-predictive - (NoTMS+Cz)/2) L vs. R	$\chi^2(1)=0.15$; $p=0.70$; $\alpha=0.00625$	$p=0.68$
	(Predictive - (NoTMS+Cz)/2) L vs. R	$\chi^2(1)=0.13$; $p=0.72$; $\alpha=0.00625$	$p=0.72$

Note: rTMS=repetitive Transcranial Magnetic Stimulation; DV=Dependent variable; SPOC=Superior Parietal Occipital Cortex; mIPS=Medial Intraparietal Sulcus; CE$_X$=horizontal interception error; IMD: initial movement direction; NoTMS=conditions without rTMS; Cz=conditions with rTMS applied to the control site Cz; UP=upward target motion; DWN=downward target motion; L=stimulation applied in the left hemisphere; R=stimulation applied in the right hemisphere.

Supplementary Table 3: rTMS effects for non-diagonal target motion on spatial movement parameters

**Site/DV**	**IN vs. OUT**	**Bootstrapped *p***
SPOC/VE$_X$	*t*(23)=0.66; *p*=0.26; *α*=0.00625; *d'*=0.13	*p*=0.50
SPOC/CE$_X$	*t*(23)=0.12; *p*=0.91; *α*=0.00625; *d'*=0.024	*p*=0.90
mIPS/VEx	*t*(23)=1.08; *p*=0.14; *α*=0.00625; *d'*=0.22	*p*=0.27
mIPS/CEx	*t*(23)=-0.10; *p*=0.92; *α*=0.00625; *d'*=-0.021	*p*=0.92
SPOC/VIMD	*t*(23)=-0.021; *p*=0.51; *α*=0.00625; *d'*=-0.0043	*p*=0.98
SPOC/IMD	χ^2(1)=0.15; *p*=0.69; *α*=0.00625	*p*=0.69
mIPS/VIMD	*t*(23)=-0.58; *p*=0.72; *α*=0.00625; *d'*=-0.12	*p*=0.55
mIPS/IMD	χ^2(1)=5.16; *p*=0.023; *α*=0.00625	*p*=0.013

Note: rTMS=repetitive Transcranial Magnetic Stimulation; DV=Dependent variable; SPOC=Superior Parietal Occipital Cortex; mIPS=Medial Intraparietal Sulcus; VE_X=variance of horizontal interception error; CE_X=horizontal interception error; VIMD: variability of initial movement direction; IMD: initial movement direction; IN=rTMS applied in the hemisphere coding the visual field in which the target moves; OUT= rTMS applied in the hemisphere coding the visual field in which the target does not move.

Supplementary Table 4: Non-predictive and Predictive rTMS effects on movement timing

Site/DV	Test	Statistics	$p_{Bootstrapped}$
SPOC/T_{ini}	Non-predictive - (NoTMS+Cz)/2	$t(23)=-2.78$; $p=0.011$; $\alpha=0.00625$; $d'=-0.57$	$p=0.0047$
	Predictive- (NoTMS+Cz)/2	$t(23)=-2.74$; $p=0.012$; $\alpha=0.00625$; $d'=-0.56$	$p=0.0062$
mIPS/ T_{ini}	Non-predictive - (NoTMS+Cz)/2	$t(23)=-2.15$; $p=0.042$; $\alpha=0.00625$; $d'=-0.44$	$p=0.027$
	Predictive - (NoTMS+Cz)/2	$t(23)=-1.67$; $p=0.11$; $\alpha=0.00625$; $d'=-0.34$	$p=0.087$
SPOC/MT	Non-predictive - (NoTMS+Cz)/2	$t(23)=0.77$; $p=0.45$; $\alpha=0.00625$; $d'=0.16$	$p=0.43$
	Predictive - (NoTMS+Cz)/2	$t(23)=1.17$; $p=0.25$; $\alpha=0.00625$; $d'=0.24$	$p=0.23$
mIPS/MT	Non-predictive - (NoTMS+Cz)/2	$t(23)=-1.07$; $p=0.29$; $\alpha=0.00625$; $d'=-0.22$	$p=0.27$
	Predictive - (NoTMS+Cz)/2	$t(23)=-0.8$; $p=0.43$; $\alpha=0.00625$; $d'=-0.16$	$p=0.42$

Note: rTMS=repetitive Transcranial Magnetic Stimulation; DV=Dependent variable; SPOC=Superior Parietal Occipital Cortex; mIPS=Medial Intraparietal Sulcus; T_{ini}=moment of movement initiation; MT=movement time; NoTMS=conditions without rTMS; Cz=conditions with rTMS applied to the control site Cz.

Supplementary Table 5: rTMS effects for non-diagonal target motion on movement timing

Site/DV	$STIM_{IN}$ vs. $STIM_{OPPOSITE}$	$p_{Bootstrapped}$
SPOC/T_{ini}	$t(23)=-1.91$; $p=0.068$; $\alpha=0.0125$; $d'=-0.39$	$p=0.050$
SPOC/MT	$t(23)=1.3$; $p=0.21$; $\alpha=0.0125$; $d'=0.27$	$p=0.18$
mIPS/T_{ini}	$t(23)=0.43$; $p=0.67$; $\alpha=0.0125$; $d'=0.088$	$p=0.66$
mIPS/MT	$t(23)=-1.44$; $p=0.16$; $\alpha=0.0125$; $d'=-0.29$	$p=0.14$

Note: rTMS=repetitive Transcranial Magnetic Stimulation; DV=Dependent variable; SPOC=Superior Parietal Occipital Cortex; mIPS=Medial Intraparietal Sulcus; T_{ini}=moment of movement initiation; MT=movement time; $STIM_{IN}$=rTMS applied in the hemisphere coding the visual field in which the target moves; $STIM_{OPPOSITE}$= rTMS applied in the hemisphere coding the visual field in which the target does not move.
